# Hybrid modeling of an ultracentrifugation process for separation of full and empty adeno-associated virus particles

**DOI:** 10.1007/s00449-024-03014-3

**Published:** 2024-05-04

**Authors:** Riccardo De-Luca, Miguel Pupo-Correia, Michael Feldhofer, Duarte L. Martins, Alexandra Umprecht, Ali Shahmohammadi, Daniel Corona, Moritz von Stosch

**Affiliations:** 1 DataHow AG, Hagenholzstrasse 111, 8050 Zurich, Switzerland; 2 DataHow Soluções de Inteligência Artificial, Unipessoal, LDA, Rua Filipe Folque 2, 1050-110 Lisbon, Portugal; 3 Baxalta Innovations GmbH, a Takeda Company, Industriestraße 67, 1221 Vienna, Austria; 4 Takeda Manufacturing U.S.A., Inc., 95 Hayden Avenue, Lexington, 02421 USA

**Keywords:** Hybrid model, Ultracentrifugation, AAV, Full/empty capsids

## Abstract

Ultracentrifugation is an attractive method for separating full and empty capsids, exploiting their density difference. Changes of the serotype/capsid, density of loading material, or the genetic information contained in the adeno-associated viruses (AAVs) require the adaptation of the harvesting parameters and the density gradient loaded onto the centrifuge. To streamline these adaptations, a mathematical model could support the design and testing of operating conditions.

Here, hybrid models, which combine empirical functions with artificial neural networks, are proposed to describe the separation of full and empty capsids as a function of material and operational parameters, i.e., the harvest model. In addition, critical quality attributes are estimated by a quality model which is operating on top of the harvest model. The performance of these models was evaluated using test data and two additional blind runs. Also, a “what-if” analysis was conducted to investigate whether the models’ predictions align with expectations.

It is concluded that the models are sufficiently accurate to support the design of operating conditions, though the accuracy and applicability of the models can further be increased by training them on more specific data with higher variability.

## Introduction

Adeno-associated virus (AAV) has become a widely used gene therapy vector, as its genetic payload (therapeutic gene) can be easily substituted to address different diseases, enabling a platform approach. Furthermore, different serotypes are used which allow targeting different cells inside the human body [1, 2]. However, during the production of the virus capsids, not all capsids are filled with the DNA insert, i.e., there are full and empty capsids (and perhaps even partially filled or double filled ones). To ensure drug product efficacy and minimize immunogenicity and toxicity, separation of full and empty and enrichment of the full capsids becomes necessary [3, 4].

One possible way of separating full and empty capsids is by ultracentrifugation, since the empty and full capsids have a difference in density [5–7]. For this purpose, the semi-purified virus material is loaded together with a density gradient medium into the ultracentrifuge, centrifuged, and then carefully harvested, such that the density gradient can be exploited for separation. Whenever the serotype or insert is changed, the density gradient medium (DGM) solutions and harvesting parameters must be adapted using insert and system scale related ranges to allow for good separation (i.e., starting from standard conditions the parameters are modified whenever serotype or insert change to obtain a good separation). The objective is to streamline the adaptation using a mathematical model describing the process behavior and to simulate the process behavior for different conditions before running a final selection experimentally, significantly reducing the experimental effort.

Models for analytical ultracentrifugation [8–10] or for nanoparticle separation [11–13] have been reported previously. These models are typically based on the momentum balance for a particle

1 $$m\cdot \frac{{d}^{2}x}{{dt}^{2}}={F}_{c}-{F}_{b}-{F}_{f},$$

with $m$ the mass of the particle, $x$ the distance of the particle to the rotation center, $t$ the centrifugal runtime, ${F}_{c}$ the centrifugal force, ${F}_{b}$ the buoyancy, and ${F}_{f}$ the reverse viscous resistance [14]. After the start-up of the ultracentrifuge, the state of uniform motion without acceleration is reached, wherefore ${F}_{c}={F}_{b}+{F}_{f}$. In principle, this equation could be used to determine the position of empty and full capsids (as well as sedimentation coefficients), but it will require knowledge about the particle masses, densities, and shapes as well as the viscosity and density of the DGM (which changes from the rotation center to the outside and with time). In the absence of this knowledge as well as the absence of measurements for different runtimes, the model proposed in the following focuses on the developed steady state, where the reverse viscous resistance (a function of particle velocity) is zero (as the particles are no longer moving), and hence, ${F}_{c}={F}_{b}$. Knowledge about the general shape of the particle distributions [10] is used to model their shape and position based on material attributes and tested process conditions. To the best of our knowledge, to date, no mathematical model for this process has been reported.

The article is structured as follows. In Sect. "Process and model", the experimental set-up and modeling methodology are described; in Sect. "Results and discussion", we present and discuss the results obtained for the proposed modeling approach and we showcase the models’ capabilities using a what-if analysis. In Sect. "Conclusions", the conclusions are presented.

## Process and model

In this section, first, the experimental set-up is described; then, the structure of the proposed modeling workflow is discussed.

### Experimental set-up

**Fig. 1:**
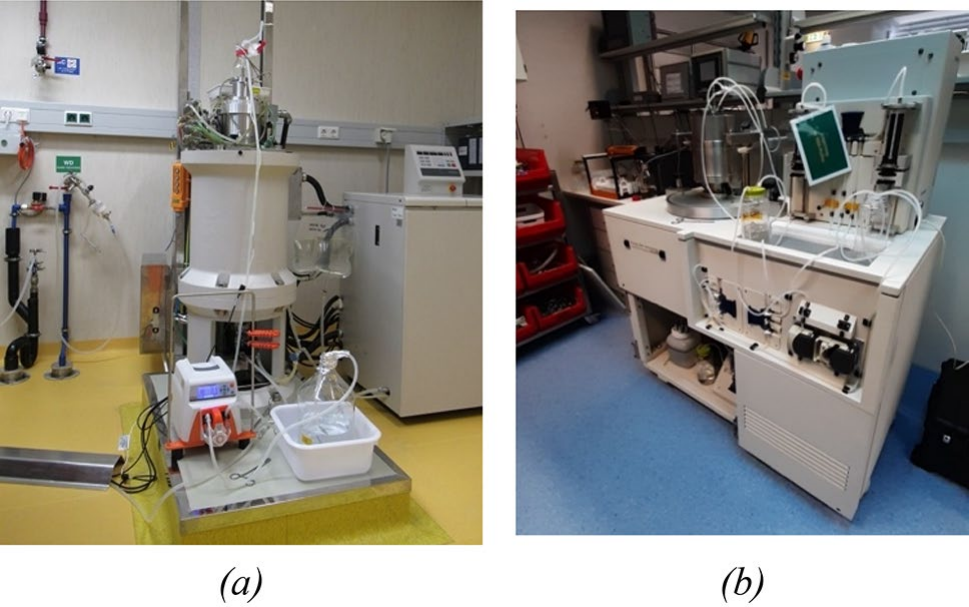
Photos of: **a** CC40S™ (Hitachi Koki); **b** AW PROMATIX 1000™ (Alfa Wassermann)

Process equipment: Two different ultracentrifugation units, CC40S™ (Hitachi Koki) and AW PROMATIX 1000™ (Alfa Wassermann), displayed in Fig. 1, were used for the process runs. The goal is to use the smaller unit for process development and then transfer the operating conditions to the larger unit and scale-up the process.

Process runs: An overview of the runs considered in this study is shown in Table 1; note that they have been performed using: (i) the two different centrifuges; (ii) three different AAV serotypes (henceforth named as “A”, “B”, “C”); and (iii) different transgene lengths (z-scores are reported for confidentiality reasons). Furthermore, Table 1 reports the information of which experiments were exploited for model training and testing.

**Table 1:** Overview of the experiments considered in this study (equipment + product features), their use in the training or test partition

Exp ID	Train/test	Equipment	Serotype	Insert length (z-scores)
#1	Train	CC40S	A	0.5132
#2	Train	CC40S	B	−1.7234
#3	Test	CC40S	B	−0.1702
#4	Train	CC40S	B	−0.1702
#5	Train	CC40S	B	−0.1702
#6	Train	CC40S	B	0.8885
#7	Test	CC40S	B	0.8885
#8	Train	CC40S	B	−0.1702
#9	Train	CC40S	A	0.4460
#10	Train	CC40S	A	0.5132
#11	Train	CC40S	B	0.9094
#12	Train	CC40S	B	0.9094
#13	Train	CC40S	B	0.9094
#14	Train	CC40S	B	0.9094
#15	Train	CC40S	B	0.8885
#16	Train	CC40S	A	0.4460
#17	Test	CC40S	A	0.4460
#18	Train	CC40S	A	0.4460
#19	Train	PROMATIX 1000	B	−1.7234
#20	Train	PROMATIX 1000	B	−1.7234
#21	Train	PROMATIX 1000	B	−1.7234
#22	Test	CC40S	B	−1.7234
#23	Train	CC40S	C	0.1842

Operation and initial conditions: The operating conditions and additional features, such as the key quality attributes of the inlet product (i.e., serotype volume/density of the loaded material, the transgene length inside the capsid), are different for each run and reported in Table 2, hereafter referred to as *Z* variables. The *Z* variables that can be manipulated are the flowrate, the volumes of the low-, mid-, high-DGM solutions. The *Z* variables that are determined by the preceding process step are volume load, density load, as well as the two OD peak areas. Those Z variables that are scale-specific were scaled using the total core volume, aiming at rendering the model scale independent.

**Table 2:** List of operating conditions and additional features, referred to as Z variables

Name	Description	u.o.m.	Scaled^a^
Flowrate	The volumetric flowrate at which the mixture is extracted from the centrifuge	L h^−1^	yes
lowDGMVol	Volume of low density DGM loaded into the centrifuge	mL	yes
midDGMVol	Volume of mid density DGM loaded into the centrifuge	mL	yes
highDGMVol	Volume of high density DGM loaded into the centrifuge	mL	yes
Initial density	Average density of all the material in the centrifuge at the start of the run (DGM solution + loaded material)	g mL^−1^	no
Vol_Load	Volume of loaded material	mL	yes
Density_Load	Density of loaded material	g mL^−1^	no
OD254_PeakArea	Peak area of the load material at 254 nm, divided by dilution factor and multiplied by the ratio of Vol_Load and volume used for measurement	AU mL	no
OD280_PeakArea	Peak area of the load material at 280 nm, divided by dilution factor and multiplied by the ratio of Vol_Load and volume used for measurement	AU mL	no
Insert length	size of the genetic payload packed into the capsid	bp [base pair]	no

^a^Here “Scaled” means that the Z variables showing “yes” are scaled by the total core volume reported for each experiment, which renders them independent of the scale of operation

Operation of the centrifuges: The centrifuge rotor is filled with the DGM as well as the process intermediate coming from the preceding step (column chromatography step). As the centrifuge accelerates, the density gradient changes from a vertical gradient to a radial gradient before reaching target speed. Subsequently, the centrifuge is operated at constant rotational speed for a given amount of time (depending on serotype and process intermediate density); both serotype-specific operating modes deliver consistent separation performance across the reported process runs. Differences in runtime can be ignored during the modeling workflow, as it is implicitly considered with the changes in serotype.

After finishing the targeted separation time, the centrifuge is decelerated, its content changes back to vertical gradient, and the material is evacuated from the centrifuge using a pump, measured by PAT systems (see Harvest measurements section) and it is automatically fractionated, hereafter referred to as harvest.

**Fig. 2:**
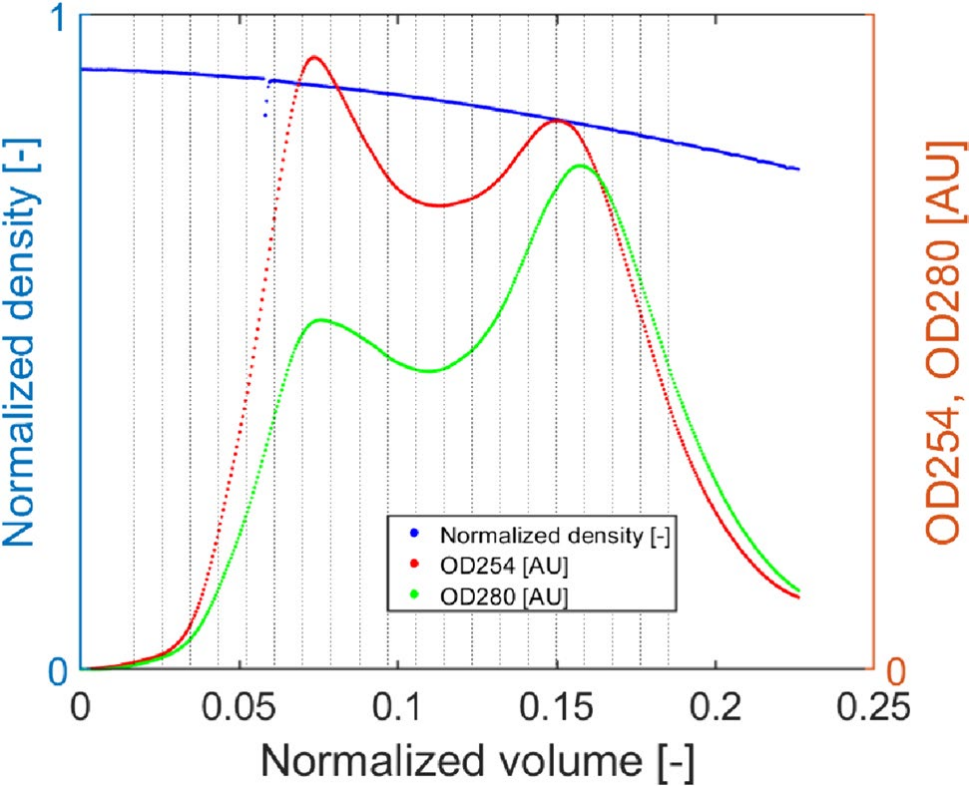
An example of the online measurements performed during an experimental run: the density gradient curve (blue solid line), and the optical density (OD) detected at 254 nm (red line) and 280 nm (green line). The peaks registered for both the two OD correspond to the volume at which the maximum amount of full and empty capsids is eluted by the ultracentrifuge, respectively; the vertical black dotted lines point out the sampling intervals of fractionation for CQA analysis and evaluation. The actual values are not explicitly reported for confidentiality reasons (color figure online)

Harvest measurements: The following measurements were recorded during the harvest of each run: (i) mixture density ($\rho$; [°Bx]); (ii) optical mixture density at two different wavelengths (${\text{OD}}_{254}$ and ${\text{OD}}_{280}$; [AU]), provided by online PAT sensors. Figure 2 shows an actual example of the profiles recorded during the harvest from one of the experimental runs. The density, measured in Brix degrees (°Bx), shows the characteristic monotonically decreasing profile of the DGM, whereas the two *OD* signals present two peaks, the first one (left) corresponding to the full AAVs and the second one (right) corresponding to the empty AAVs. Note that the distance between the peaks can vary (up to the point where the two peaks overlap into one) depending on the chosen operating conditions, and the more the peaks are distant from each other, the more efficient the separation between the target product and the product-derived impurities (empty AAVs).

Critical quality attributes: As can be seen in Fig. 2, fractions with predefined size have been collected during the harvesting process with an automated system at preset time intervals using dedicated harvest pump, and offline measurements on each product fraction were performed to evaluate the following Critical Quality Attributes (CQAs): (i) genome-containing AAV particles presence measured by polymerase-chain-reaction (PCR [vg mL^−1^]), (ii) full/empty ratio measured by analytical anion exchange chromatography (F/E [−]), and (iii) AAV capsid presence measured by enzyme-linked immunosorbent assay (ELISA [cp mL^−1^]).

### Model development

**Fig. 3:**
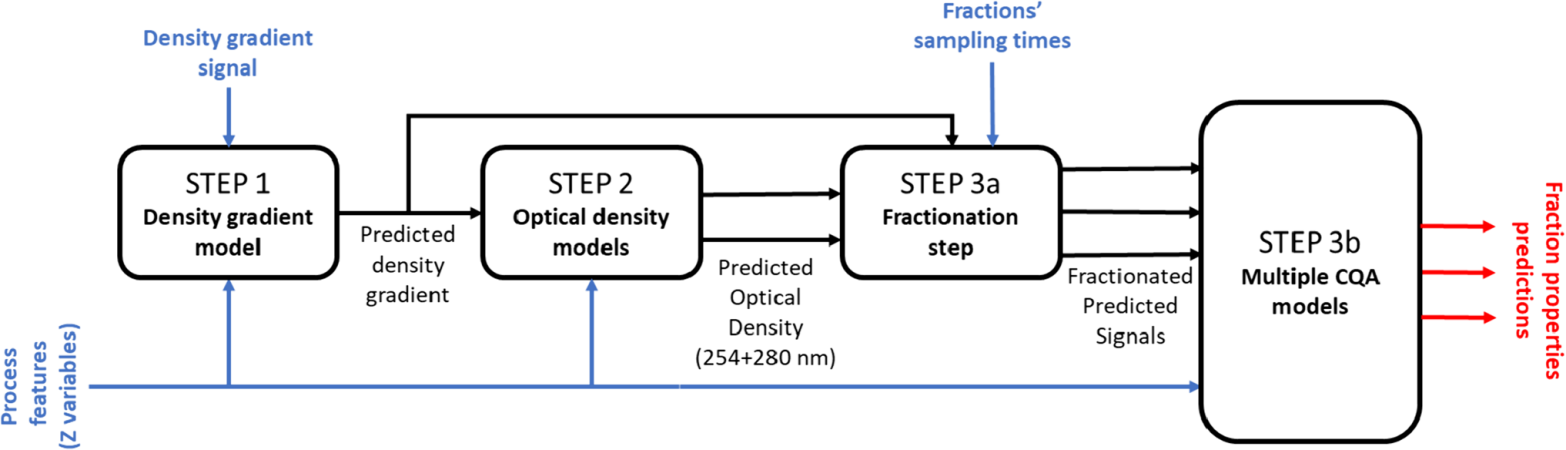
Schematic view of the modeling interactions and interplay

The experimental data described in the previous paragraph were used to build, in a sequential way,^1^ the following models: (i) density gradient model; (ii) optical density models (one at 254 nm, one at 280 nm); (iii) CQA models (to describe PCR titer, full-to-empty capsid ratio (F/E ratio), ELISA titer), which we found to be most efficient and whose interlinks are schematically shown in Fig. 3.

#### Step 1: the density gradient model

**Fig. 4:**
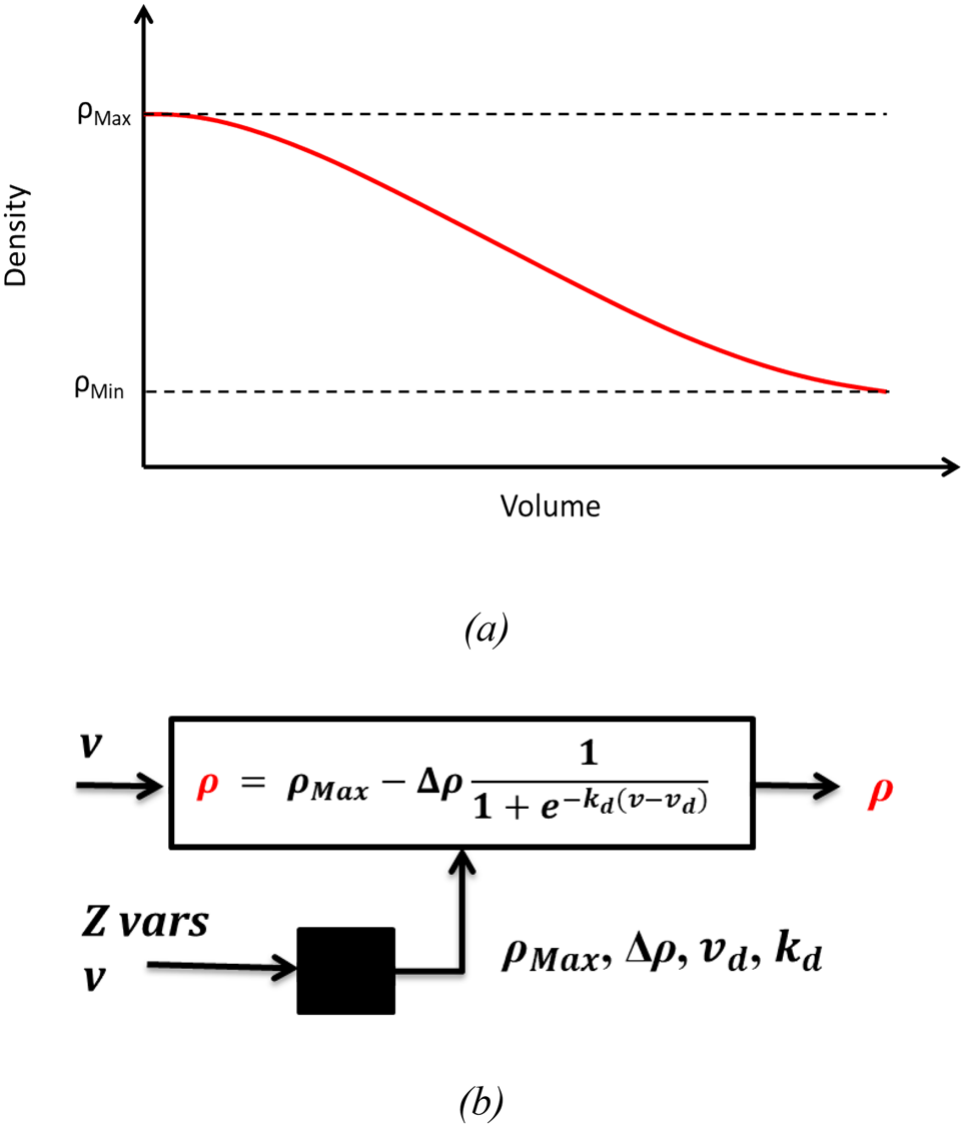
**a** Qualitative representation of the inverse sigmoid function used to approximate the density profile. **b** Block diagram. The white block represents the semi-empirical function used to describe the density (ρ), whereas the black block represents the data-driven modeling step for unknown parameters’ estimation

The purpose of using this model is to describe the density gradient during harvesting after the separation procedure. It assumed that a steady state was reached during the centrifugal separation, i.e., acceleration and velocity of the particles in radial direction is zero, wherefore buoyant and centrifugal force for the particles are equal. Also, the density gradient is assumed to be fully developed and prolongation of the runtime would not result in changes in the density gradient. We assumed that the density $\rho$ [g cm^−3^] profile can be approximated by an inverse sigmoid function (see Fig. 4a) and investigated the appropriateness of this function for the reported runs prior to this study. The function is:

2 $$\rho = {\rho }_{Max}-\Delta \rho \frac{1}{1+{e}^{-{k}_{d}(v-{v}_{d})}}$$

where ${\rho }_{{\text{Max}}}$ [g cm^−3^] is the maximum density (registered at the beginning of the process), $\Delta \rho { = }\rho_{{{\text{Max}}}} { - }\rho_{{{\text{Min}}}}$ [g cm^−3^] is the difference between the maximum density and the density at the end of the experiment, ${v}_{{\text{d}}}$ [−] is the normalized evacuated volume corresponding to the sigmoid curve’s point of inflection, and ${k}_{{\text{d}}}$ [−] is the function decay coefficient.

In this study, the values of the parameter set **θ**_ρ =_ [${\rho }_{Max}$, $\Delta {\uprho }$, ${v}_{d}$, ${k}_{d}]$ have been estimated by a feedforward artificial neural network (NN) which is function of the *Z* variables reported in Table 2 and the normalized evacuated volume $v$ (as reported in Fig. 4b). Note that the input values for the NN were normalized by subtracting the mean and dividing with the standard deviation of the Z variables in the training data. The NN architecture consists of a single hidden layer with tangential hyperbolic activation functions (nodes). The nodes of the input and output layers are linear. The number of nodes in the hidden layer was systematically varied and the “optimal” number was selected as the one with the smallest architecture presenting a sufficiently low RMSE on the training data. The weights of the NN were identified by: (i) minimizing a weighted least square loss function for the training set using a stochastic gradient descent approach which was initialized 40 times from random values and (ii) choosing from the 40 initialization the 10 models performing best on the training set, which were for subsequent predictions aggregated, yielding a bootstrap-aggregated hybrid model.

The hybrid model and its analytical derivatives were implemented in Matlab® R2022a. Due to the “static” nature of the model, the computations could be accomplished using matrix computations. The computational time for training of a bootstrap-aggregated hybrid model with a fixed number of nodes in the hidden layer took about 15 min on an AMD Ryzen™ 9 5900HX processor with 64.0 GB RAM.

#### Step 2: the optical density models

**Fig. 5:**
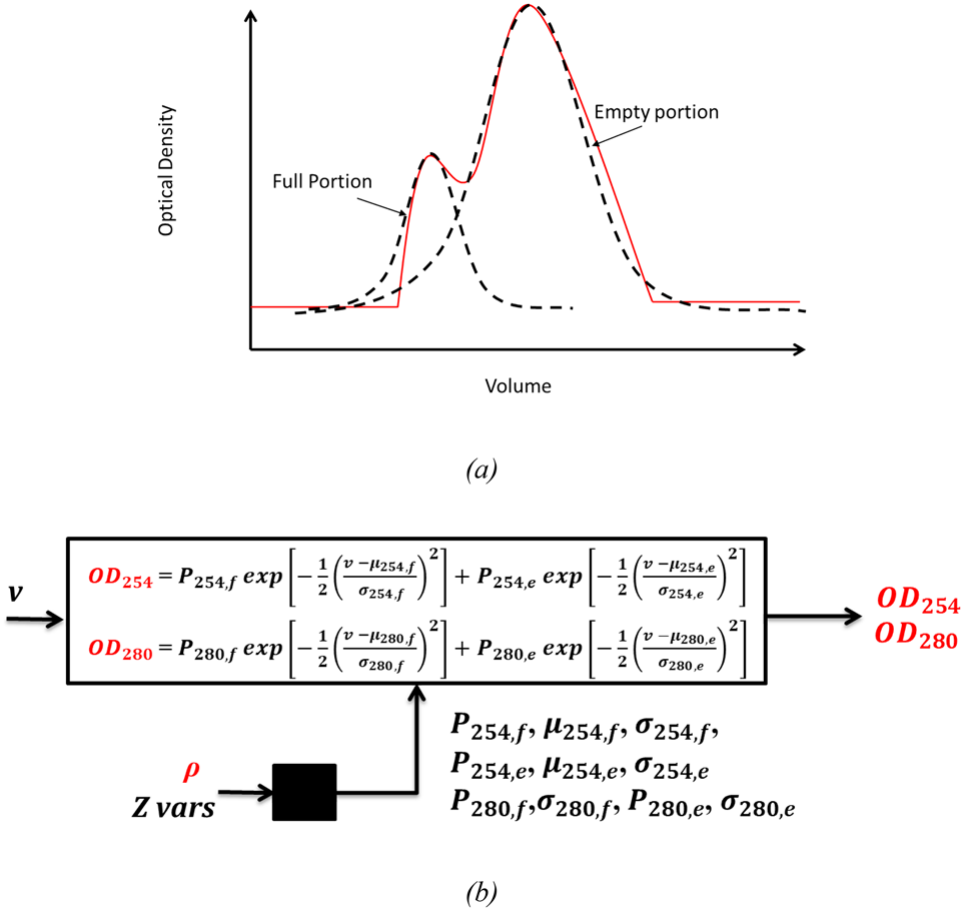
**a** Qualitative representation of the function used to approximate the particle distributions observed through the optical density profile at 254 and 280 nm. **b** Block diagram. The white block represents the semi-empirical function used to describe the optical density profile at 254 and 280 nm), whereas the black block represents the data-driven modeling step for unknown parameters’ estimation

This model describes the distributions of full and empty capsid particles after separation, assuming that the distributions have a Gaussian function shape and that buoyant and centrifugal force for the particles are equal at harvest. The distributions can be observed through the optical density profiles at 254 nm and 280 nm during harvest. Hence, both optical density profiles are approximated by a second degree mixed Gaussian function as reported in Fig. 5; in practice, we assumed that: (i) both 254 nm and 280 nm signals can be described as two superimposed Gaussian functions representing the evacuation of full capsids (first peak) and empty capsids (second peak); (ii) the position of the two peaks is approximately located at the same elution volume for both 254 nm and 280 nm optical density signals. The final functions are expressed as:

3 $${OD}_{254} = {P}_{254,f} \mathit{exp}\left[-\frac{1}{2}{\left(\frac{v -{\mu }_{254,f}}{{ \sigma }_{254,f}}\right)}^{2}\right]+{P}_{245,e}\mathit{exp}\left[-\frac{1}{2}{\left(\frac{v -{\mu }_{254,e}}{{ \sigma }_{254,e}}\right)}^{2}\right]$$

4 $${OD}_{280} = {P}_{280,f}\mathit{exp}\left[-\frac{1}{2}{\left(\frac{v -{\mu }_{280,f}}{{ \sigma }_{280,f}}\right)}^{2}\right]+{P}_{280,e}\mathit{exp}\left[-\frac{1}{2}{\left(\frac{v -{\mu }_{280,e}}{{ \sigma }_{280,e}}\right)}^{2}\right]$$

In Eq. 3, ${P}_{254,f}$[AU] and ${P}_{254,e}$ [AU] represent the peak magnitude of the OD at 254 nm related to products with full and empty capsids, respectively; ${\mu }_{254,f}$ [−] and ${\mu }_{254,e}$ [−] are the values of normalized evacuated volume at which peaks occur for full and empty capsids; and ${\sigma }_{254,f}$ [−] and ${\sigma }_{254,e}$ [−] are the standard deviations of the two Gaussian curves (a similar description applies for Eq. 4, referring to 280 nm optical density). Note that, due to assumption (ii) described in this paragraph, we have ${\mu }_{254,f}= {\mu }_{280,f}$ and ${\mu }_{254,e}= {\mu }_{280,e}$, so the vector of unknown parameters is **θ**_OD =_ [${P}_{254,f}$, ${\mu }_{254,f}$, ${\sigma }_{254,f}$, ${P}_{254,e}$, ${\mu }_{254,e}$, ${\sigma }_{254,e}$, ${P}_{280,f}$,${\sigma }_{280,f}$, ${P}_{280,e}$, ${\sigma }_{280,e}$].

Similarly to density model’s parameters, the values of the parameter set **θ**_OD_ are the output of a neural network (NN); in this case, the NN is function of: the *Z* variables reported in Table 2 and the density values predicted by the density gradient model, as shown in the block diagram in Fig. 5b; NN architecture consists again of a single hidden layer of hyperbolic tangential nodes and the optimal number of nodes, weights, and bootstrap-aggregated hybrid model structure was determined as described before for the density gradient model. The optical density hybrid model was implemented in Matlab® R2022a in the same fashion as the density gradient model, taking about the same amount of time for training.

#### Step 3: CQA models

This step consists of two sub-steps (see Fig. 3): (Step 3a) preprocessing of predicted profiles from both density gradient model and optical density models; (Step 3b) model calibration.

**Fig. 6:**
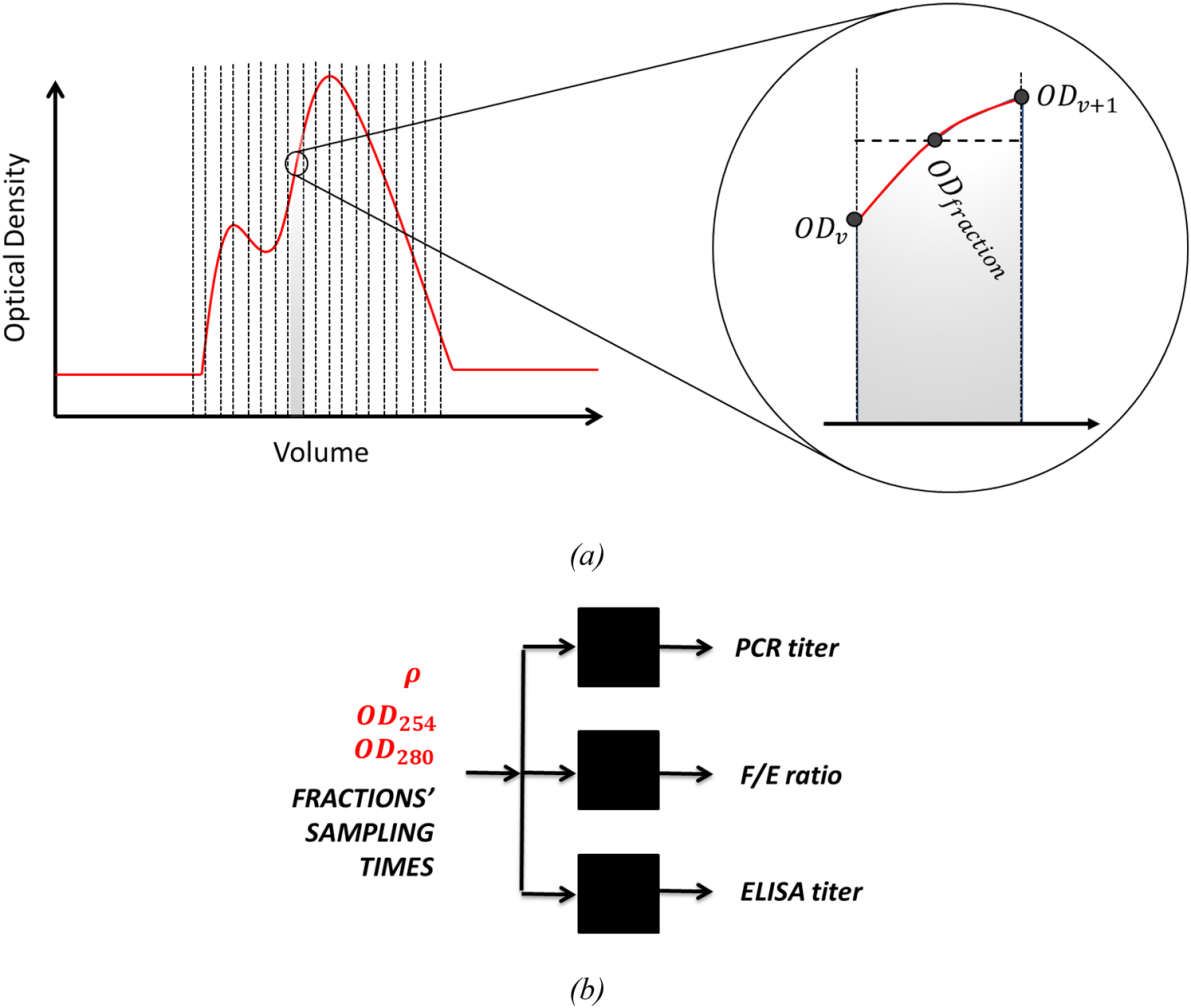
**a** Qualitative representation of the preprocessing substep (Step 3a) to calculate the fraction values for the optical density profiles predicted in Step 2. Note that the same procedure is applied to the density gradient profile predicted in Step 1. **b** Block diagram. The black blocks represent the data-driven modeling approach used to estimate CQAs from the fraction values of optical density and density gradient calculated in (Step 3a)

Step 3a: During the preprocessing step, the predicted signals of density, optical density at 254 nm, and optical density at 280 nm are exploited to calculate their average value corresponding to each fraction extracted during harvest; in practice (see Fig. 6), given the values of the evacuated volumes (*v*_*i*_ and *v*_*i*+1_) between two fractions (*i* and *i* + 1), we estimated the value of the desired predicted feature *y*_frac,i_ as:

5 $${y}_{frac,i} = \text{ } \frac{1}{{v}_{i+1}-{v}_{i}}\underset{i}{\overset{i+1}{\int }}ydv.$$

Step 3b: During the calibration step, the preprocessed data (i.e., the calculated fractions values of density gradient, OD254, OD280) and the insert length values are used as input to train a simple feedforward neural network model for each CQA (see Fig. 6b). Note that, in this case, we adopted a split ratio 90:10 for fraction measurements train/validation sets generation. Furthermore, we used square root transformation of the raw CQAs’ values, which helped to “normalize” their distributions, originally characterized by positive skewness.

After training, all models were evaluated on the test set partition (see Table 1) as well as an additional data set comprising 2 runs, referred to as blind set. All computations were performed in Matlab® R2022a on an AMD Ryzen™ 9 5900HX processor with 64.0 GB RAM. Density gradient and optical density models were calibrated using an implementation of the Adam optimization algorithm [15] with a learning rate of 1e-3 and 1000 iterations, whereas *fitcrnet* Matlab® internal function was used to fit the three CQA models.

## Results and discussion

In this section, we first present and discuss the model performance in terms of goodness-of-fit; then, we evaluate the model prediction performance through two additional runs, exploring unknown zones of the design space. Finally, we perform simulations to assess the validity of model predictions from a physical point of view when perturbing key operating conditions.

### Density gradient model

**Fig. 7:**
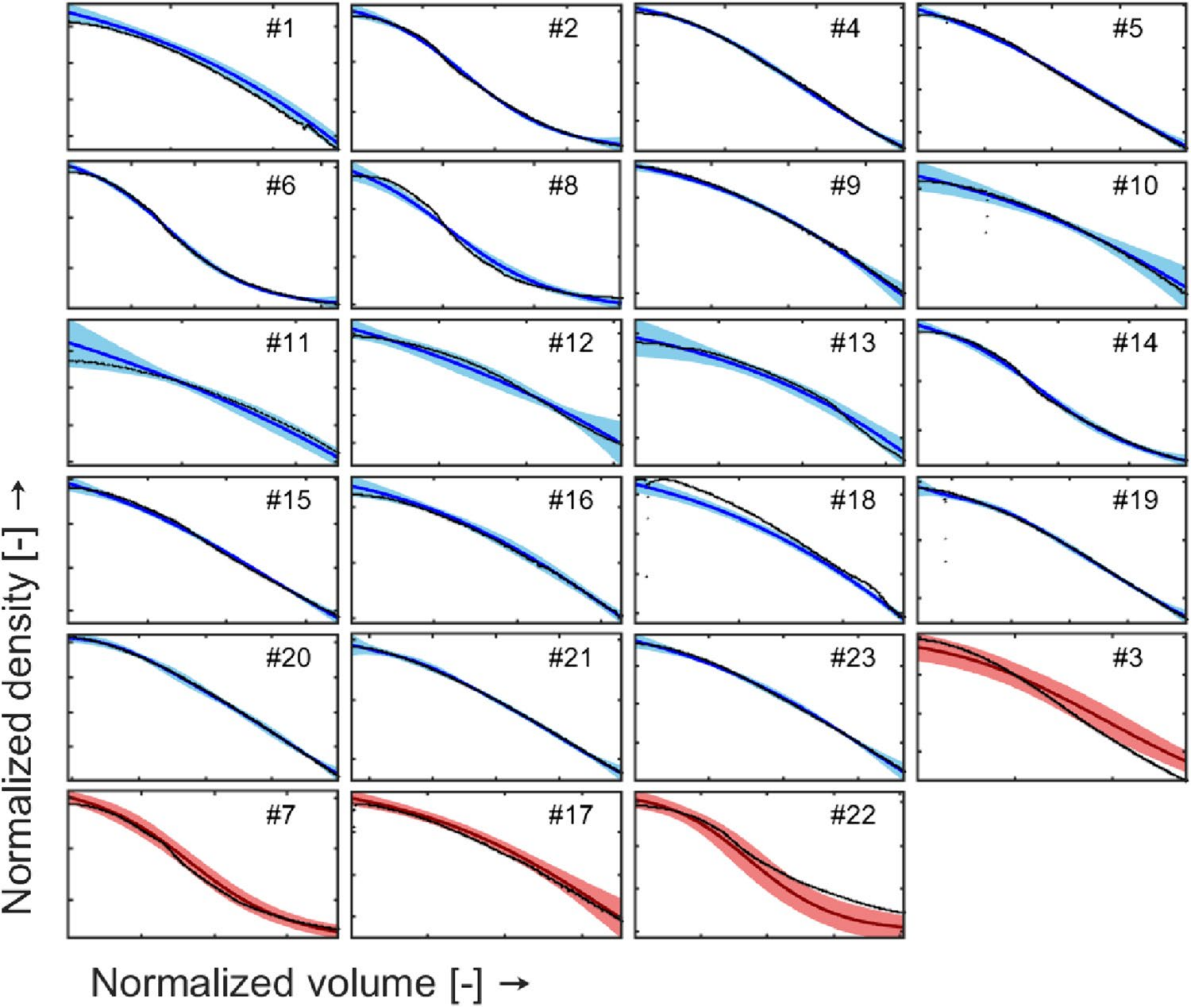
Measured (black points) and predicted density gradient values (blue solid line for training data and red solid line for test data) over evacuated volume. The shaded area represents the prediction interval of the model (color figure online)

**Fig. 8:**
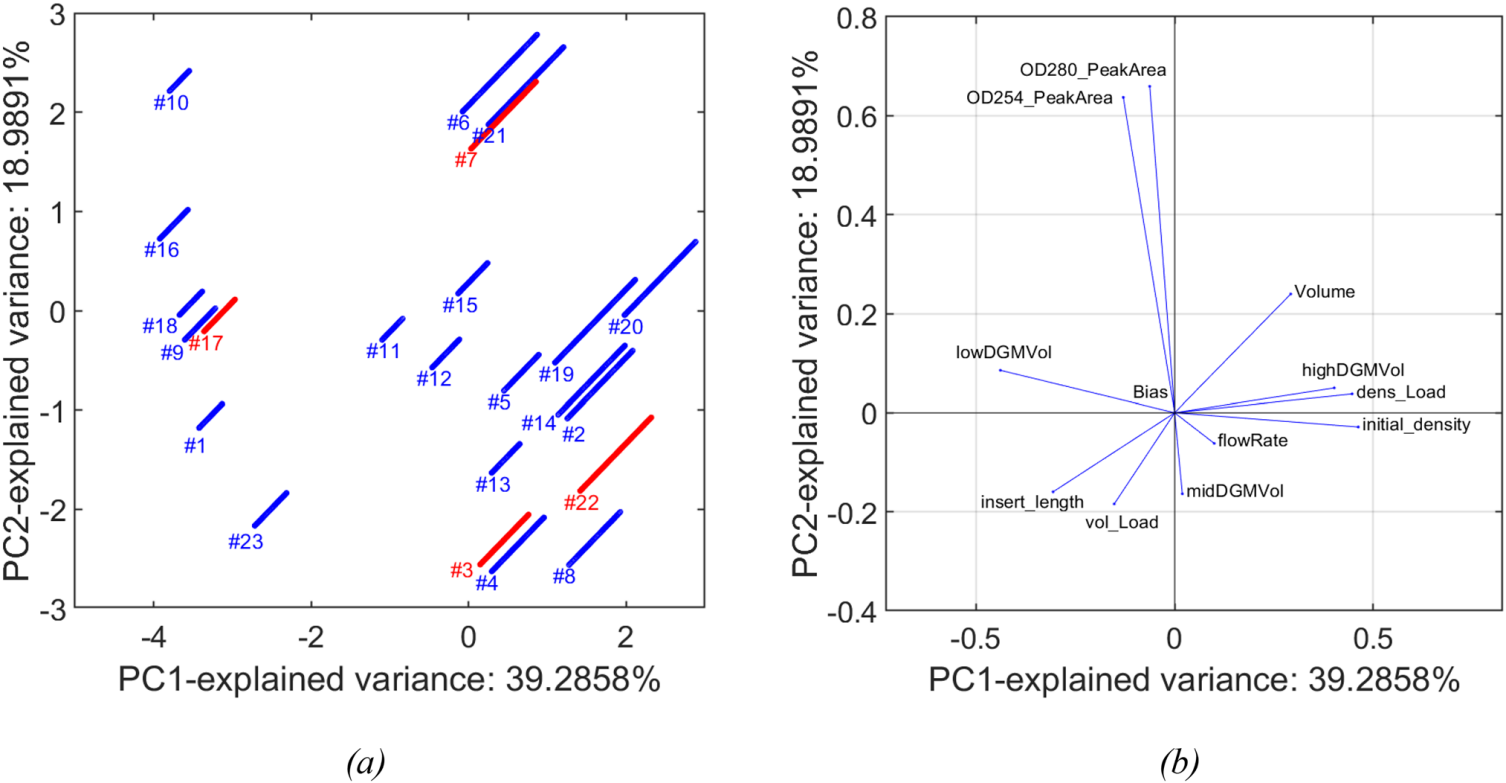
PCA analysis on NN input variables for the train and test runs. **a** Scores plot (blue and red points represent the samples related to train and test data, respectively; grouped by batch); **b** loadings plot. Note that the exploited features for PCA are the Z variables and the normalized volume, since they are the inputs to the neural network used to model the density gradient (color figure online)

The predicted and measured density gradient values are shown over the evacuated volume in Fig. 7. Furthermore, we show the scores of each batch when projected by the first and second principal component using Principal Component Analysis (PCA, see Fig. 8a). Inspection of the predicted profiles show that all density gradient profiles are well modeled, although some runs show higher uncertainty in prediction; in particular:

- Training runs #10, #11, #12, #13 show more uncertainty both at the start and the end of the run. This seems to be explained by the runs’ conditions as visually apparent from Fig. 8, i.e., #10 is the run performed at the highest OD_PeakAreas, whereas runs #11, #12, and #13 are performed using high insert length values (see Table 1 for reference to the z-scores).
- Test runs #3 and #22 show both higher discrepancy between measured and predicted values, especially during the second half of the run; as becomes apparent from Fig. 8a, the behavior on these runs can be explained by the position of these two batches in the score plot, far from the origin and distant from other batches more than the other two test runs (#3 and #17).

The position and width of the full and empty capsid peaks can be expected to be directly impacted by the density gradient, for conditions where the buoyancy and centrifugal forces are equal (see Eq. 1). Hence, the gradient density model performance will also impact that of the optical density model (as can be seen in case of run 22 or for the what-if scenarios described below). In light of this, it seems particularly important to assess the model predictions in combination with the relative run position in the PCA score plot. It seems that those runs of the test set, which can be observed to be particularly close in the score plot, exhibit low prediction errors. However, the density gradient of run 22 which falls in between the scores of runs #2, 4, and 8 is not as well predicted. While overall, the performance of the density gradient model is very satisfactory in terms of goodness-of-fit and prediction performance on the test set, the predictions for conditions that exhibit PCA scores that are distant from those that the model was trained on, should be assessed with care.

### Optical density models

**Fig. 9:**
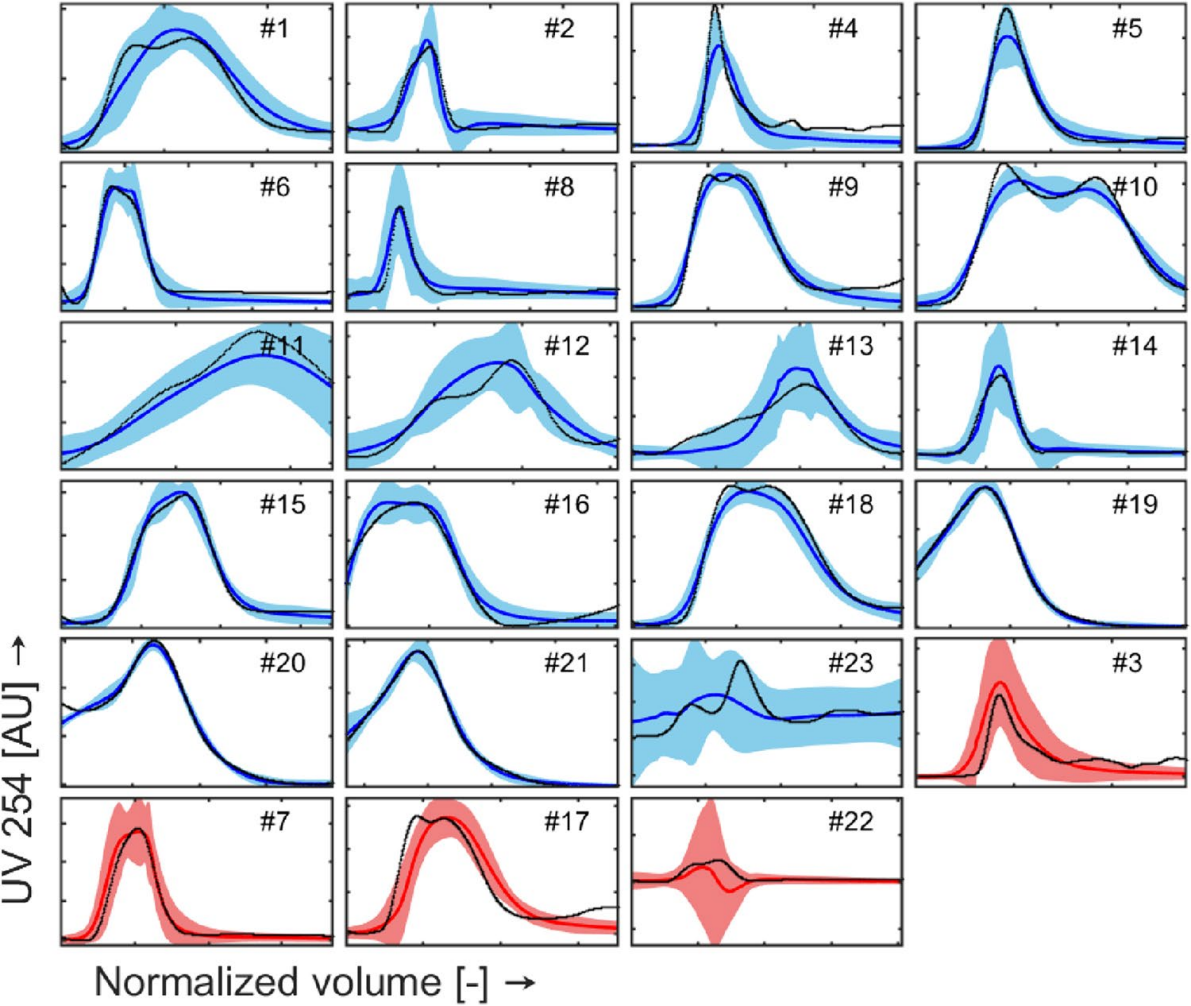
Measured (black points) and predicted OD254 values (blue solid line for training data and red solid line for test data) over evacuated volume. The shaded area represents the prediction interval of the model (color figure online)

**Fig. 10:**
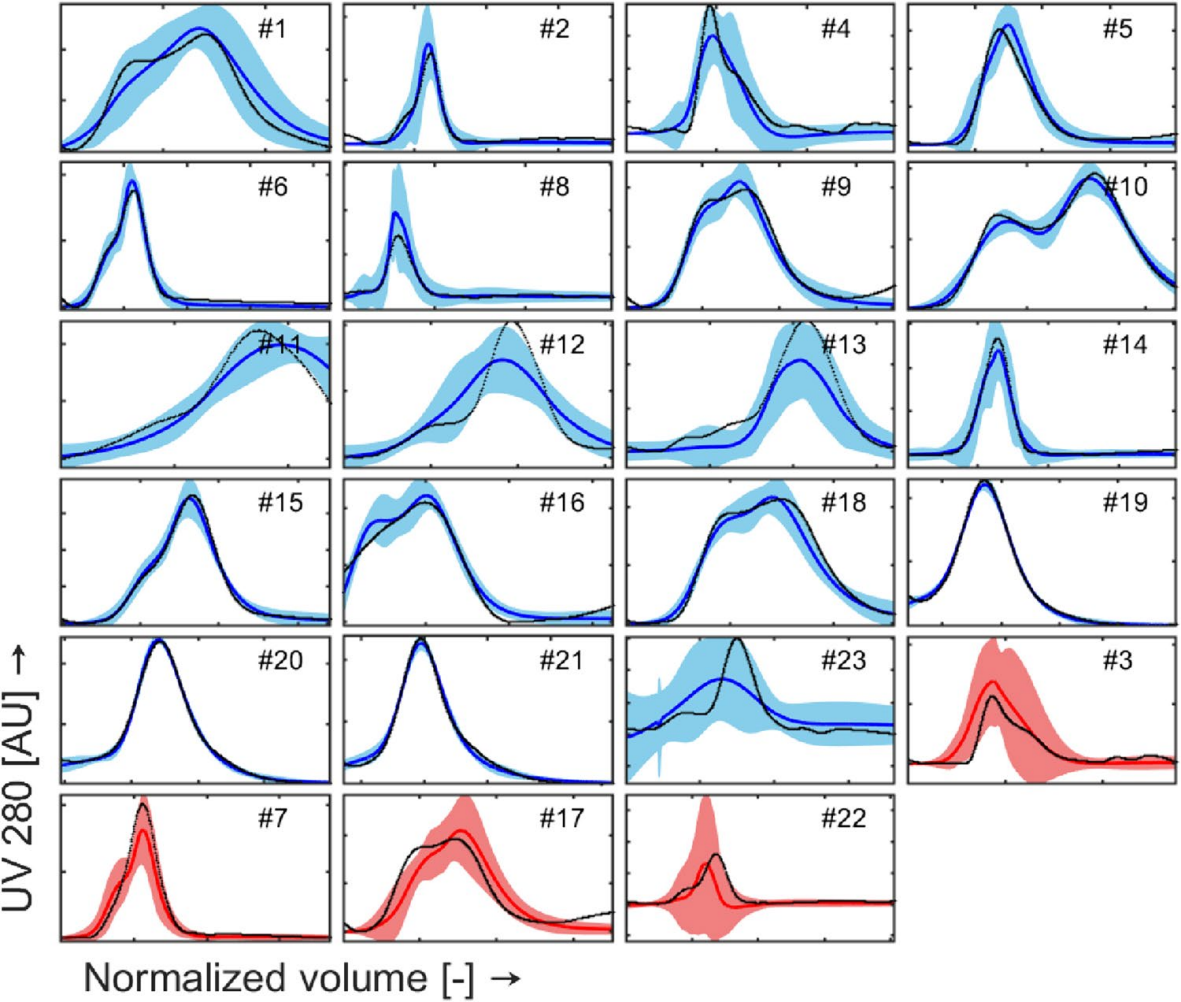
Measured (black points) and predicted OD280 values (blue solid line for training data and red solid line for test data) over evacuated volume. The shaded area represents the prediction interval of the model (color figure online)

The model predictions for *OD*_254_ and *OD*_280_ are shown in Fig. 9 and Fig. 10, respectively. Inspection of the plots shows that:

- The model can predict the peak magnitude for all the experimental runs.
- The prediction intervals contain the actual profiles in their entirety for more than 94% of the runs; nevertheless, the identification of the zone of eventual split between full and empty particles (peaks separation) is still not performed for all the runs.
- The uncertainty in density gradient prediction (see §3.1) reported for exp. #3, #11, #12, and #13 propagates to optical density models, as demonstrated by the breadth of the prediction intervals in Fig. 9 and Fig. 10. Nevertheless, the actual trends are properly described by the predicted profiles.
- Run #23 (and #1, to a lesser extent) shows high prediction uncertainty; as shown in Fig. 8a-b, experiment #23 is performed at high lowDGMVol values but low OD_PeakAreas (around 1 order of magnitude of difference with respect to all the other runs performed at similar lowDGMVol values).
- Test runs are predicted in a satisfactory way. Runs #3 and #22 present higher prediction uncertainty: this result could have been expected given the marginal positions of these two runs in the scores plot reported in Fig. 8.

The predicted positions of empty and full capsid peaks by the two optical density models should help to understand whether the process conditions (before running the experiment) should be tweaked to achieve a better separation of the peaks. In light of this objective, the prediction of the relative position of the mean and the width of the peaks is important. Though it can be seen that the prediction of the exact shape of the two peaks could be significantly improved, the width of the peak(s) is very well captured, allowing to understand the separation performance. Hence, overall, the performance of the two optical density models is satisfactory in terms of goodness-of-fit and prediction performance. A further step in model refinement could be to perform additional runs in the unknown design space regions to reduce the model prediction uncertainty and improve peak separation modeling, and/or to weight the residuals in the loss function in a different way.

### CQA models

**Fig. 11:**
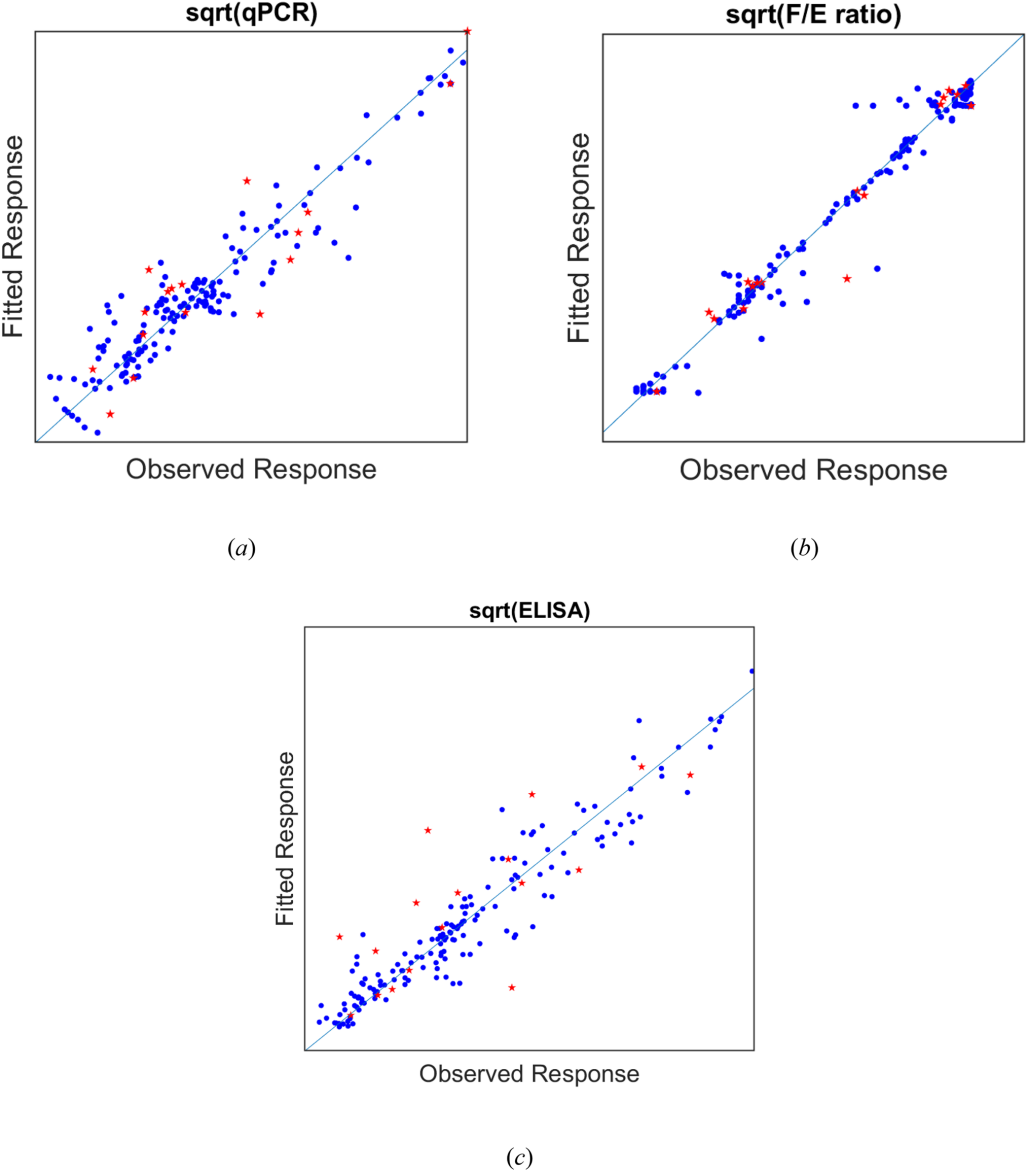
CQA models. Predicted vs measured data for: **a** qPCR, **b** F/E ratio, **c** ELISA (blue samples for training data and red samples for test data). Actual values are not reported for confidentiality reasons (color figure online)

The fitting ability of the 3 CQAs models can be assessed in Fig. 11a, b, and c. We can see that PCR and ELISA titers (Fig. 11 a, c) do not exhibit significant bias, whereas F/E ratio (Fig. 11b) shows some underpredictions at lower amount of F/E ratio and some overpredictions at higher values; this behavior can be due to the uncertainty in optical density peaks’ predictions propagating to F/E ratio model. However, these data correspond to only around 10% of the total samples, whereas the model provides very accurate performance for all the other samples. It is interesting to note that the prediction performance of the optical density models, which could be improved with respect to the prediction of the exact shape of the peak(s), does not seem to significantly deteriorate the prediction performance of the CQA models. This might be due to the fact that: 1) an integration is performed on the predicted optical density (which might level out over- and underpredictions); and 2) the overall width and position of the peaks is captured. Hence, the models’ predictions can be used to judge the separation performance and over, the models’ performance are acceptable, as long as the run conditions are not too different as discussed for the gradient density model.

### Design space exploration

**Fig. 12:**
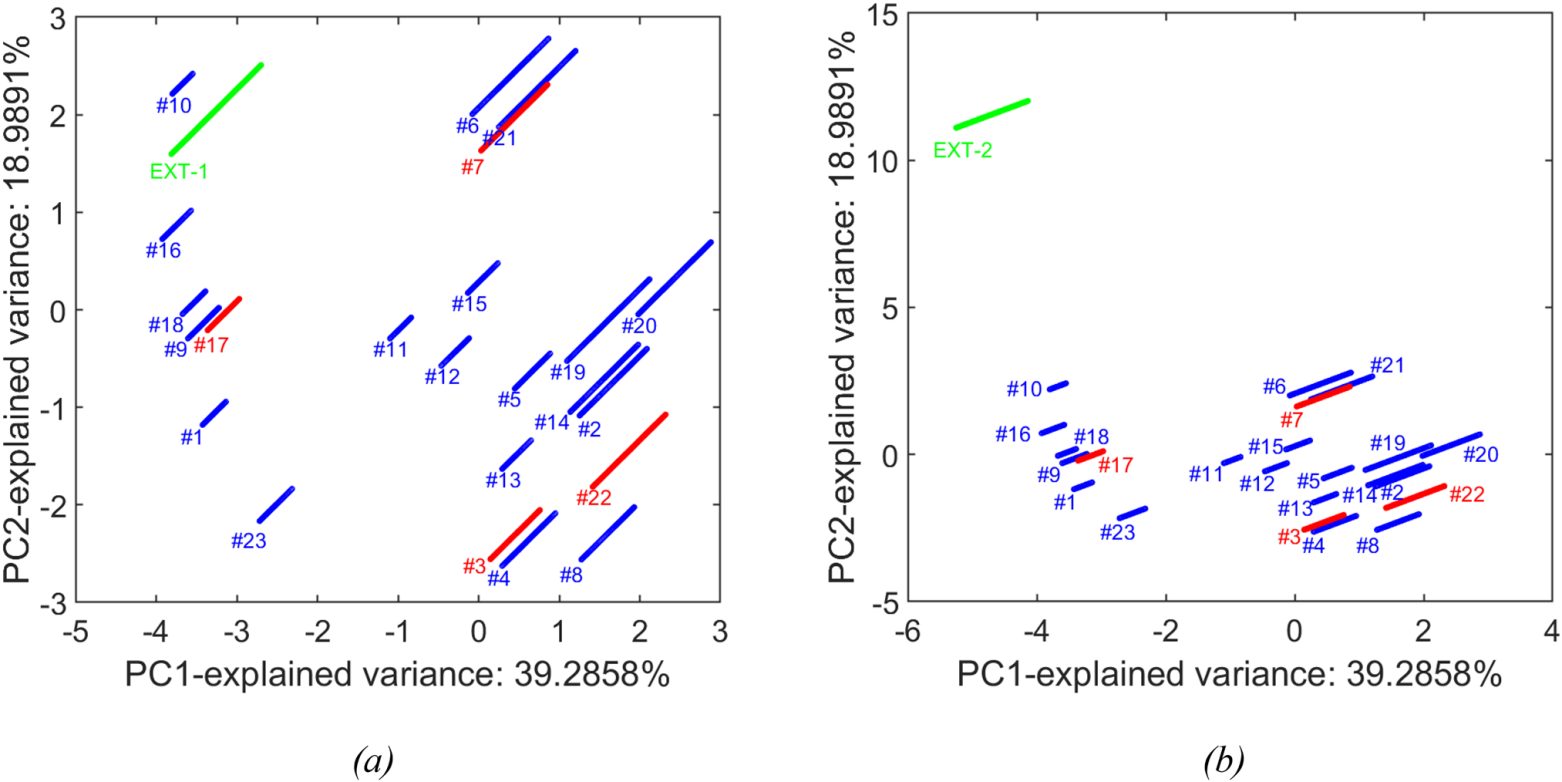
Projection on the space generated by the first and second principal component using PCA analysis on density gradient model’s NN input variables of: **a** run EXT-1 and **b** run EXT-2. Note that the exploited features for PCA are the Z variables and the normalized volume and the loadings are the same reported in Fig. 8b

The prediction performance of density gradient and optical density models was evaluated on two additional blind runs, with the aim to explore different zones of the design space, henceforth named as *EXT-1* and *EXT-2*. We report the scores of each batch when projected by the first and second principal component using PCA (see Fig. 12) to get qualitative information about the relative position of each run with respect to the runs used for model training/test. We see that run *EXT-1* was performed at operating conditions quite similar to the ones used to train the existing models, whereas run *EXT-2* is far apart from the space origin; in particular, *EXT-2* marginal position is due (see Fig. 8b) to significantly higher values of OD254_PeakArea/OD280_PeakArea than the ones reported for all the other runs.

**Fig. 13:**
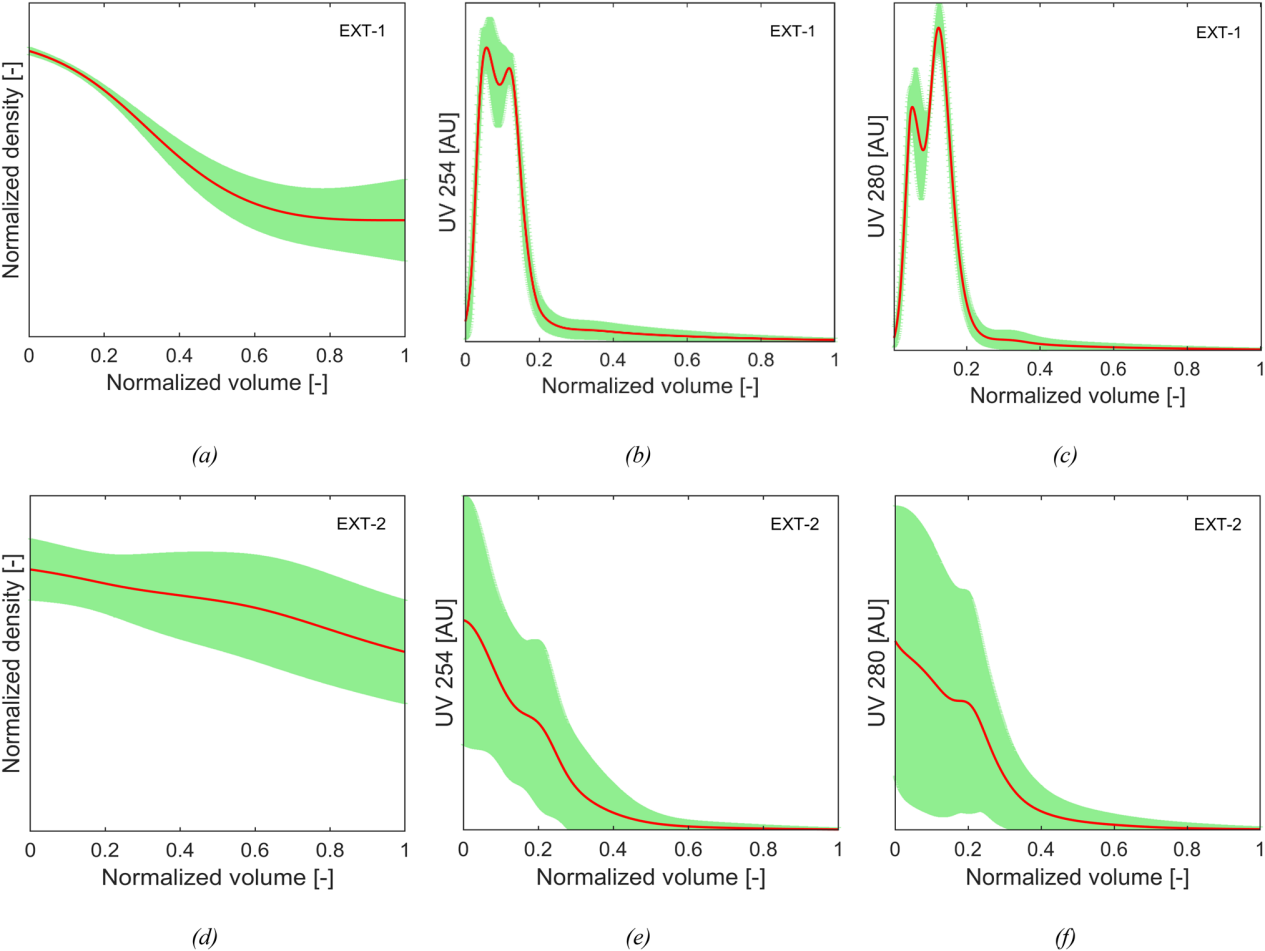
Predictions of normalized density and optical density for: **a, b, c** run EXT-1, **d, e, f** run EXT-2. The red solid lines represent the predicted profiles, whereas the shaded green areas represent the prediction interval throughout the run (color figure online)

The predicted values for the two runs are reported in Fig. 13. The comparison between the results leads to the conclusion that the trained models exhibit less uncertainty in prediction for *EXT-1* (Fig. 13a, b, c) than for *EXT-2* (Fig. 13d, e, f), since the trained models’ performance can be expected to decrease in case of extrapolation from the training conditions. Moreover, the uncertainty on density gradient prediction increases with the normalized evacuated volume for both runs. This result is likely to be related to the low number of runs used for model training with levels of OD254_PeakArea/OD280_PeakArea comparable to *EXT-2.*

### What-if analysis

Defining the quality parameters and operating conditions adopted for run *EXT-1* as “base case”, we performed the following “what-if analysis” tasks, to compare the simulated profiles when altering the volume of midDGM solution loaded into the centrifuge (*midDGMVol* case study).

We compared the predictions obtained using the actual midDGMVol for *EXT-1* with the ones obtained after simulating 4 different scenarios, generated by modifying the actual midDGMVol (from −50% to + 50% of the original value). Note that, in each scenario, a corresponding increase/reduction of highDGMVol was implemented to obtain the same initial density.

**Fig. 14:**
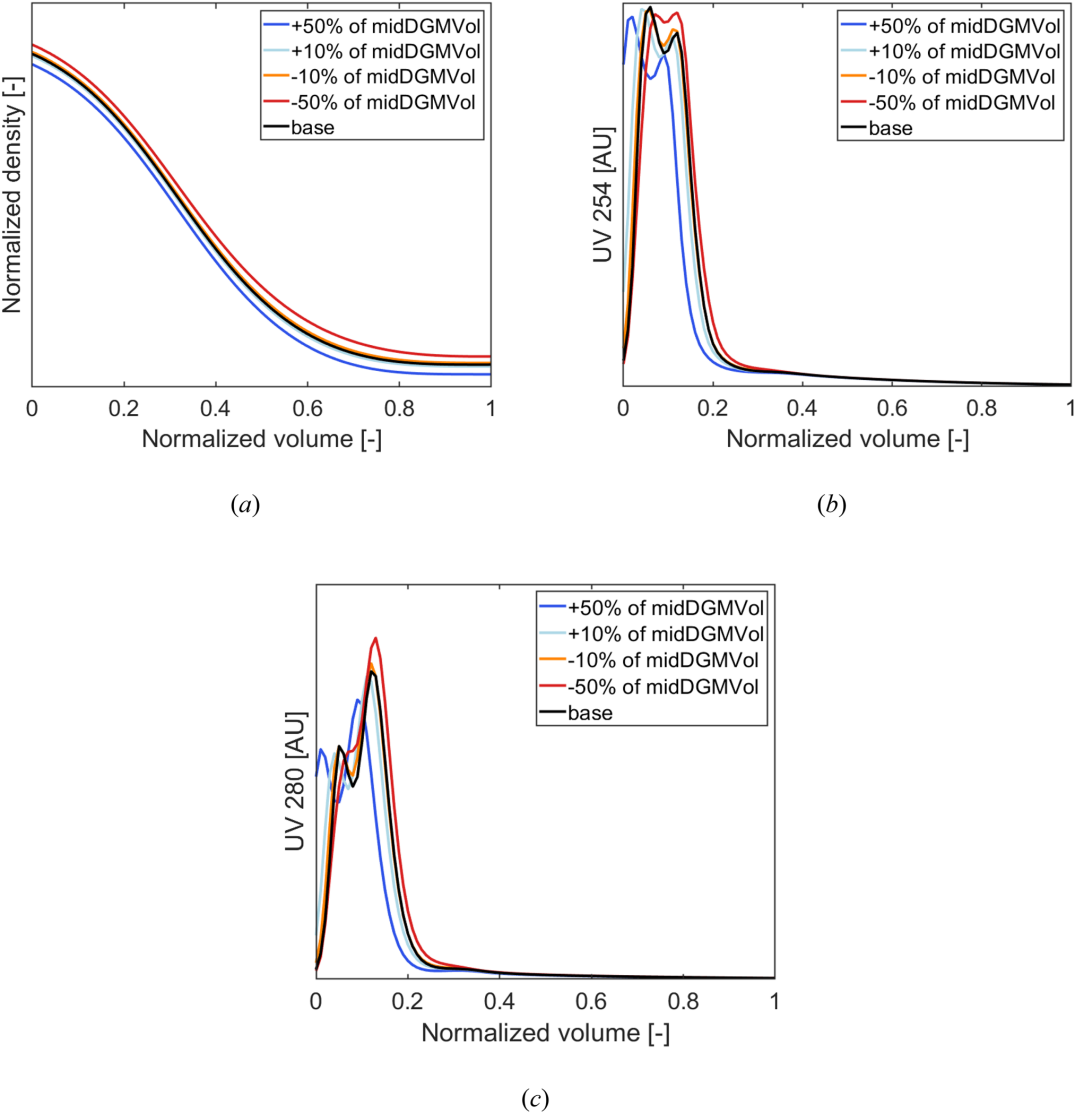
What-if analysis: midDGMVol case study. Predicted profiles of: **a** normalized density, **b** optical density at 254 nm, and **c** optical density at 280 nm

The results, reported in Fig. 14, show that the higher the quantity of midDGMVol (lower highDGMVol), the lower the gradient start density, and the more shifted to the left the optical density profiles. As the overall DGMVol would be lower, wherefore the gradient start density as well as the overall evolution would be shifted to left, this is in line with what is expected. Moreover, peaks’ separation in optical density (see Fig. 14b and c) is less detectable when reducing midDGMVol. As the width and the position of the peaks are directly impacted by the density gradient, a less pronounced slope of the density gradient will result into a less good separation of the particles. Hence, the observations of the “what-if” analysis are in line with the expected behavior, but no experimental runs were performed to further check the predictions.

## Conclusions

In this project, we proposed a novel modeling approach to describe separation of full and empty virus capsids by ultracentrifugation, with main attention on density gradient and optical densities prediction reliability. The main results obtained from this study were the following:

1. A trained set of linked models that can describe: (i) the evolution in time of the gradient density and full and empty capsid particle distributions as observed by optical density (OD), as well as (ii) Critical Quality Attributes (CQA) values, just exploiting a reduced number of key operating conditions and raw material quality information.
2. A valuable solution in case of scale-up across different ultracentrifugation units and change of formulated product (different serotypes/different size of genomic payload packed into the capsids).

The proposed models were trained and tested with real experimental data, showing good prediction fidelity on average. The results from the “what-if” analysis underpin the physical meaning of models’ predictions despite the hybrid nature of each model. Further steps in models’ refinement could be obtained using more experimental data at best performed at different conditions (as an example, loaded capsid particle material for which high OD Peak Area values are observed); finally, defining a more precise way to identify the actual starting point of the harvest (and, if needed, recording measurements till the end of the harvest) could further enhance the current model performances and reduce prediction uncertainty due to signal shifting.

In principle, the model could be transferred for the modeling of other ultracentrifuge applications, perhaps helping to streamline the separation development of nanoparticles, gene vectors, or other micro-carriers.

## Footnotes

1 As the optimal density model depends on the density gradient estimation and as the multiple CQA model depends on the estimation of the optical density and density gradient models, the models were built successively starting from the density gradient model.
